# Supplementary material for: Evaluation of Disability Progression in Multiple Sclerosis via Magnetic-Resonance-Based Deep Learning Techniques
Source: Int J Mol Sci. 2022 Sep 13;23(18):10651. doi: 10.3390/ijms231810651 (PMC9505100; doi:10.3390/ijms231810651)
Supplement: Supplementary file 1 [file ijms-23-10651-s001.zip › ijms-1879125-supplementary.pdf]

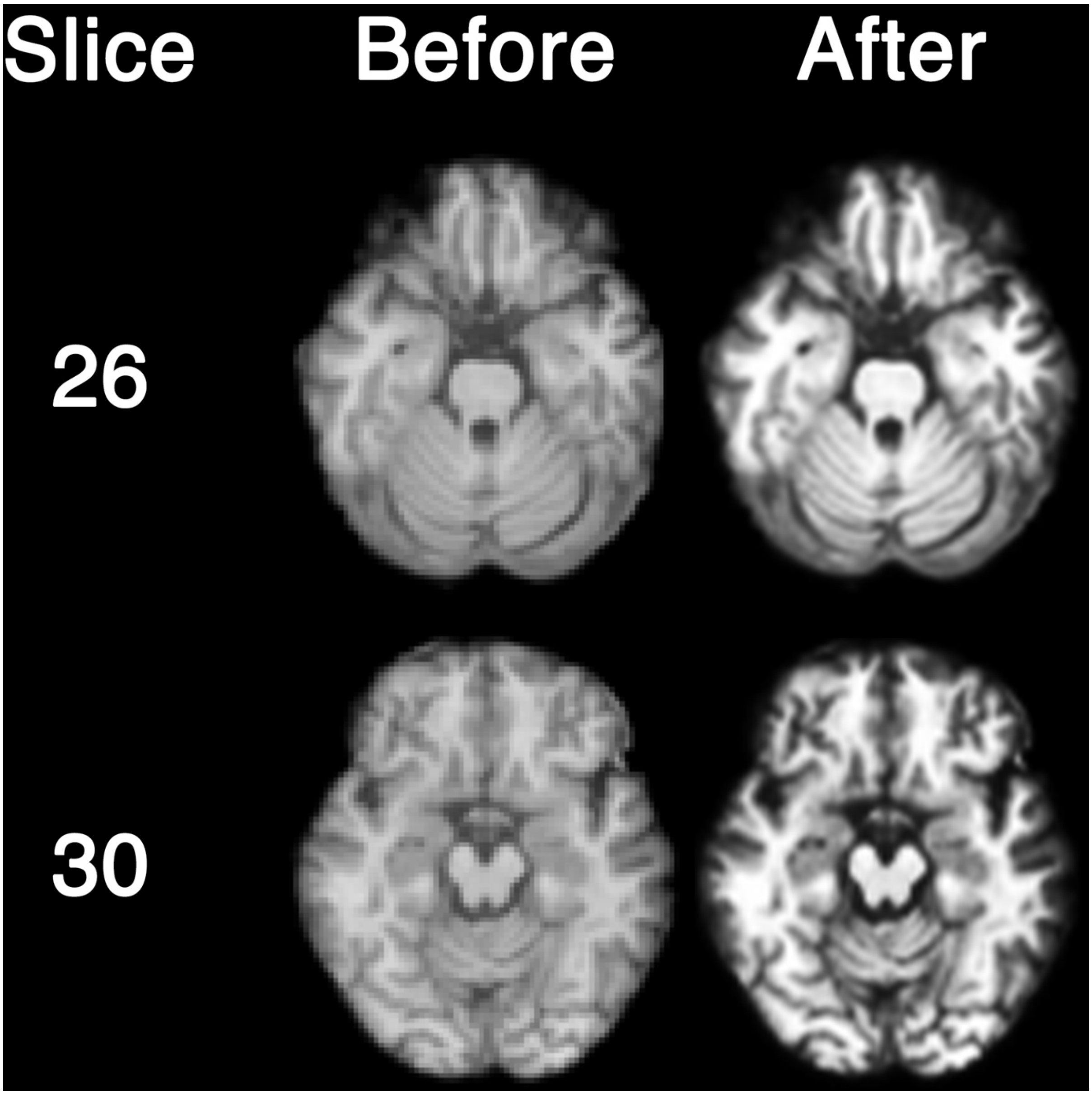

Figure S1. Contrast-maximized image. Two examples of slice image before and after having applied the homemade code to maximize contrast. From one subject, we randomly selected slice 26 and 30 for display purpose.
